# Parental History of Childhood Maltreatment and Offspring Attachment Insecurity and Disorganization: Two Meta-Analyses

**DOI:** 10.1177/15248380241282995

**Published:** 2024-10-01

**Authors:** Aino Elina Sirparanta, Camille Danner Touati, Chantal Cyr, Raphaële Miljkovitch

**Affiliations:** 1 Laboratoire Paragraphe EA 349, Université Paris 8, France; 2 UR CLIPSYD, Université Paris Nanterre, France; 3 Université du Québec à Montréal, Canada; 4 Institut Universitaire Jeunes en Difficulté, CIUSSS Centre-Sud-de-l’Île-de-Montréal, Canada

**Keywords:** child abuse, attachment, intergenerational transmission of trauma

## Abstract

Research findings have shown that parental history of childhood maltreatment (CM) increases the risk of insecure and disorganized attachment in offspring. However, the extent of the detrimental effects of childhood trauma on attachment in the next generation is unclear. The current meta-analyses aimed at synthesizing the available literature on the link between parental history of CM and offspring attachment insecurity and disorganization (with no restriction of offspring age). In total, 25 studies (23 unique samples; *N* = 2,592) comprising *u* = 61 effect sizes were included. Offspring age ranged from 12 to 79 months (*M*_weighted_ = 18.69; *SD*_weighted_ = 11.53). Findings from two three-level random effects meta-analyses revealed a weak but significant combined effect of parental history of CM on child attachment insecurity (*k* = 20, *u* = 35, *r* = .06) and a non-significant effect on child attachment disorganization (*k* = 12, *u* = 26, *r* = .03). For the meta-analysis on disorganization, effect sizes were weaker in more recent studies, and trim and fill analyses provided evidence of publication bias. These findings provide a nuanced view of the intergenerational transmission of childhood trauma phenomenon, whereby parents’ self-reported history of CM does not appear decisive for child attachment. Conclusions could not be drawn for specific types of CM because of the small number of studies. Research with more objective measures of parental exposure to CM is needed to gain a more comprehensive view of the possible intergenerational effects of CM on child attachment.

## Introduction

Childhood maltreatment (CM) represents a global and widespread phenomenon (see Stoltenborgh et al., 2015 for meta-analysis on worldwide prevalence rates). It is associated with deleterious mental health outcomes in childhood (e.g., Maclean et al., 2019; Winter et al., 2022) and adulthood (e.g., see Baldwin et al., 2023; for meta-analysis). Besides CM’s detrimental effects on the victim, it also affects the victim’s relationship with their children by altering subsequent parenting behaviors (see Savage et al., 2019 for meta-analysis). However, studies on parental history of CM and offspring attachment reveal mixed findings. Although some studies point to direct negative effects on child attachment (Galbally et al., 2022; Lyons-Ruth & Block, 1996), others suggest that parental exposure to CM may only be linked to child attachment when indirect specific pathways are taken into account (Ahlfs-Dunn et al., 2022; Alto et al., 2021), or at particular levels of specific moderating variables (Ludmer et al., 2018). To gain a clearer understanding of the association between the parental history of CM and the quality of offspring attachment, we conducted two meta-analyses, one on attachment insecurity and another on attachment disorganization.

### Child Maltreatment

CM is defined as any type of abuse (i.e., physical, emotional, or sexual) or neglect that an individual is exposed to before 18 years of age (World Health Organization, 2023). Research suggests that the detrimental effects of CM are likely to be carried over into adulthood. For example, adults exposed to CM show an increased risk of mental health problems (e.g., see Baldwin et al., 2023 for meta-analysis).

In support of Fraiberg et al.’s (1975) initial work, describing alterations in the development of mother–child bonds in dyads with a maternal history of CM, the detrimental effects of CM are not limited to the victims themselves, as it also impacts the relationship they develop with their children. Maternal history of CM is associated with less optimal parenting behaviors, namely, less positive and more negative parenting, and more difficulties in the mother–child relationship (see Savage et al., 2019 for meta-analysis). Furthermore, a history of CM could, to some extent, alter parents’ ability to effectively appreciate their children’s mental states (i.e., parental reflective functioning; see van Rensburg et al., 2023 for systematic review). Given that sensitive parenting (see De Wolff & Van IJzendoorn, 1997; Madigan et al., 2024; Verhage et al., 2016; Zeegers et al., 2017 for meta-analyses) and parental reflective functioning (see Zeegers et al., 2017 for meta-analysis) are associated with child attachment security, it seems that parental history of CM is likely to lead to insecure attachment in the next generation.

### Parental History of CM and Offspring Attachment

According to the attachment theory, an infant’s attachment system is activated in stressful circumstances (e.g., being far from the caregiver or alarmed), which prompts them to initiate attachment behaviors (e.g., such as crying) aimed at increasing proximity with the caregiver and at restoring a sense of security (Bowlby, 1969). Children adapt their attachment behaviors according to the quality of their caregiver’s responses to maximize parental investment (e.g., Main, 1990). Thus, variation in parental caregiving likely results in variations in child attachment behaviors (Ainsworth et al., 1978; Main, 1990). For example, in the Strange Situation Procedure (Ainsworth et al., 1978), which assesses child-to-parent attachment following brief separation–reunion episodes, securely attached infants seek proximity and are easily soothed by their caregiver upon the reunion. Insecure-avoidant infants tend to ignore their caregiver and to minimize the expression of attachment needs, whereas insecure-resistant infants not only maximize distress signals and actively seek proximity to the caregiver but also angrily resist contact (Ainsworth et al., 1978). Finally, disorganized children exhibit behaviors indicating a lack of organized strategy for engaging with their caregiver: in the caregiver’s presence, they appear disoriented, confused, or fearful and can display contradictory or incomplete behaviors (Main & Solomon, 1986). Studies have found increased rates of disorganized attachment in samples concerned with maltreatment or cumulative socioeconomic risks (see Cyr et al., 2010 for a meta-analysis). Identifying factors likely to impact infant or child attachment quality is important, given that attachment insecurity and even more so disorganization are recognized as significant risk factors for mental health problems during childhood (see Dagan et al., 2021; Fearon et al., 2010; Madigan et al., 2016 for meta-analyses) and later in life (e.g., Sroufe et al., 2005).

Over the years, it has been widely accepted that parental history of CM has a negative impact on the development of attachment organization. However, recent inconsistent results suggest a less straightforward picture, pointing to a need for further investigation and a finer understanding. In their seminal study, Lyons-Ruth and Block (1996) found that insecure children of mothers with histories of childhood violence were more likely to be disorganized than those of mothers without such histories. However, the association between the severity of maternal childhood trauma and offspring attachment security was not significant (Lyons-Ruth & Block, 1996). These findings suggest that maternal exposure to CM could impact attachment (dis)organization rather than attachment (in)security.

Subsequent studies have examined the effect of parental CM on offspring attachment in different ways, yielding mixed findings. In their recent meta-analysis on the distributions of infant attachment classifications, Madigan et al. (2023) found 67% and 39% of insecure and disorganized children, respectively, in samples with parental CM (*k* = 6), compared to 48% and 23% in samples without parental CM (*k* = 279). However, although the differences between the two groups appear large, they were not statistically significant.

Other studies have pointed to indirect associations between maternal history of CM and child attachment insecurity. Ahlfs-Dunn et al. (2022) reported no direct link between maternal history of CM and infant attachment insecurity, but only an indirect link via disrupted prenatal maternal representations of the child. Similarly, Alto et al. (2021) found no direct link between maternal history of CM and child attachment disorganization, but an indirect one through maternal depression and sensitivity. Parental history of CM may also impact child attachment via parent’s attachment state of mind, as coded with the Adult Attachment Interview (George et al., 1985). Namely, CM history can lead to the development of an “unresolved” attachment state of mind (i.e., lapses in the monitoring of reasoning or discourse while discussing loss or a traumatic event; Bailey et al., 2007; Byun et al., 2016; George et al., 1985; Main et al., 2002). And when parents are “unresolved,” their children are more likely to exhibit disorganized attachment (see Van IJzendoorn, 1995; Verhage et al., 2016 for meta-analyses). Hence, by contributing to an unresolved state of mind in the parent, parental exposure to CM could indirectly lead to child attachment disorganization. However, given that the unresolved classification also includes unresolved loss, the specific contribution of the parent’s unresolved past abuse in child attachment disorganization is not clearly determined.

Yet other studies suggest conditional associations. Ludmer et al. (2018) reported maternal genetic and hormonal characteristics to moderate the association between maternal history of CM and child attachment disorganization. While not explicitly examining moderator effects, Granqvist et al. (2014) found maternal history of CM to be significantly correlated with child attachment disorganization and marginally correlated with attachment insecurity in dyads where the mothers were diagnosed with intellectual disability, but not in control dyads. Likewise, Kwako et al. (2010) reported an association between maternal childhood sexual abuse and child insecurity in school age but not in infancy or preschool age. Even though this difference could be due to divergent measures of attachment for younger versus older children, it could also point to a moderating effect of offspring age. Furthermore, Gerlach et al. (2022) reported findings that suggest a differential susceptibility of boys and girls to different family risk factors. In their study, distal risk factors, which included caregiver’s experience of CM, were associated with greater attachment insecurity in girls. Parental history of CM could, hence, differently impact attachment in female and male offspring. Finally, Galbally et al. (2022) reported a positive association between maternal history of childhood sexual abuse and insecure-avoidant child attachment, whereas the links between other types of maternal CM and child attachment were not significant. This suggests that the association between parental CM and offspring attachment may vary according to the type of CM under focus.

In sum, although relatively mixed, findings point to a potential negative impact of parental history of CM on offspring attachment. However, the actual strength of this association is not clearly determined.

### Potential Substantive and Methodological Moderators

In addition to offspring gender and age, and the type of CM, other sample characteristics and methodological features of the studies may also account for the potential heterogeneity among study findings. Regarding sample characteristics, parent gender may also moderate effect sizes. Although most studies on parental history of CM and offspring attachment have included mother–offspring dyads, findings from a systematic review of studies on Holocaust survivors by Dashorst et al. (2019) indicate that mothers’ trauma history is more strongly associated with offspring’s mental health problems than fathers’ trauma history. The authors proposed that this could be partly due to the fact that mothers are most often the primary caregivers (e.g., see Musick et al., 2016) and that women exposed to childhood trauma can be particularly vulnerable to stress during pregnancy, which could, in turn, impact the child even before birth (Taouk & Schulkin, 2016). Different sample risk characteristics may also moderate the association between parental history of CM and offspring attachment. First, CM and socioeconomic risks are associated with more insecure and disorganized child attachments (see Cyr et al., 2010; Madigan et al., 2023 for meta-analyses). Hence, parental history of CM could more strongly impact offspring attachment when the offspring is also exposed to CM and in the presence of more sociodemographic risks. Second, marginally higher rates of child attachment disorganization and marginally lower rates of security were found by Madigan et al. (2023) in samples with parental psychopathology. Parents cumulating both a history of CM and psychopathology could be particularly likely to have children with insecure and disorganized attachments.

Methodological moderators, such as the type of assessment of CM or attachment, may also impact the association between parental history of CM and offspring attachment. Both parental exposure to CM and offspring attachment can be measured as categorical or continuous variables. Information on parental history of CM can be obtained from external informants (e.g., case files), be reported by participants themselves, or by multiple informants. Likewise, assessment of attachment can be based on the coding of external observers, on caregiver reports of child attachment behaviors, or on participant self-reports. Moreover, prospective and retrospective measures of CM can be used. In addition, attachment can be assessed with behavioral, representational, or questionnaire measures, and the measured construct may differ accordingly: a general trait (e.g., state of mind with respect to attachment) or a relationship-specific measure. All these assessment characteristics may impact effect sizes and should be examined as potential moderators.

Moreover, other study features could also contribute to heterogeneity between study findings. First, given that the accuracy with which studies may estimate an association is likely to vary as a function of the adequacy of the research methods implemented, study quality could moderate effect sizes (e.g., Feeley, 2020). Especially, using unreliable measures can lead to measurement error and bias the combined effect size toward zero (Wiernik & Dahlke, 2020). Hence, it is essential to consider the quality of each included study as a potential moderator, notably regarding the validity and reliability of CM and attachment measures. Second, as child welfare and social welfare policies and practices as well as legislations with regard to CM differ from one country to another, detection and intervention practices may differ greatly. As a result, victims of CM may find assistance more easily in some countries compared to others. Therefore, study country should be examined as a moderator. Finally, the way CM is perceived (Spilsbury et al., 2018), defined, and responded to (Gilbert et al., 2012) has changed over the years. These changes may make it easier to disclose and detect CM, allowing intervention with a larger proportion of CM victims. This could, in turn, impact the findings of studies on the intergenerational effects of CM. Hence, examining publication year of the included studies as a potential moderator appears important. This also allows to examine whether evidence of a “decline effect” is present (Schooler, 2011). The decline effect refers to the fact that reported effect sizes seem to diminish with time; this can be due to publication bias, where initial findings are published only when the observed effects are large, whereas subsequent studies are published even when weaker effects are found (Schooler, 2011).

### The Current Study

Taken altogether, studies suggest a potential negative impact of parental history of CM on offspring attachment. However, they have yielded mixed findings and point to the potential influence of substantive and methodological moderators on this link. To gain a better understanding of this association, the primary aim of the present study was to synthesize the available research data on the association between parental history of CM and offspring attachment insecurity and disorganization. In this study, CM is defined as comprising physical, sexual, and emotional/psychological abuse, as well as neglect. We expected positive combined correlations (*r* effect sizes) between parental history of CM and offspring attachment insecurity and disorganization. Furthermore, we aimed to examine whether publication bias was present and whether substantive and methodological indicators moderated effect sizes.

## Methods

### Transparency and Openness

We adhered to the Meta-Analytic Reporting Standard guidelines for meta-analytic reporting (Appelbaum et al., 2018). All meta-analytic data and analysis code relevant to this study are available at https://osf.io/7pjnr/?view_only=3e5f454f25b54df4b933ff79646f31e8 (link anonymized for review). This study was not preregistered.

### Literature Search

**Figure 1.**
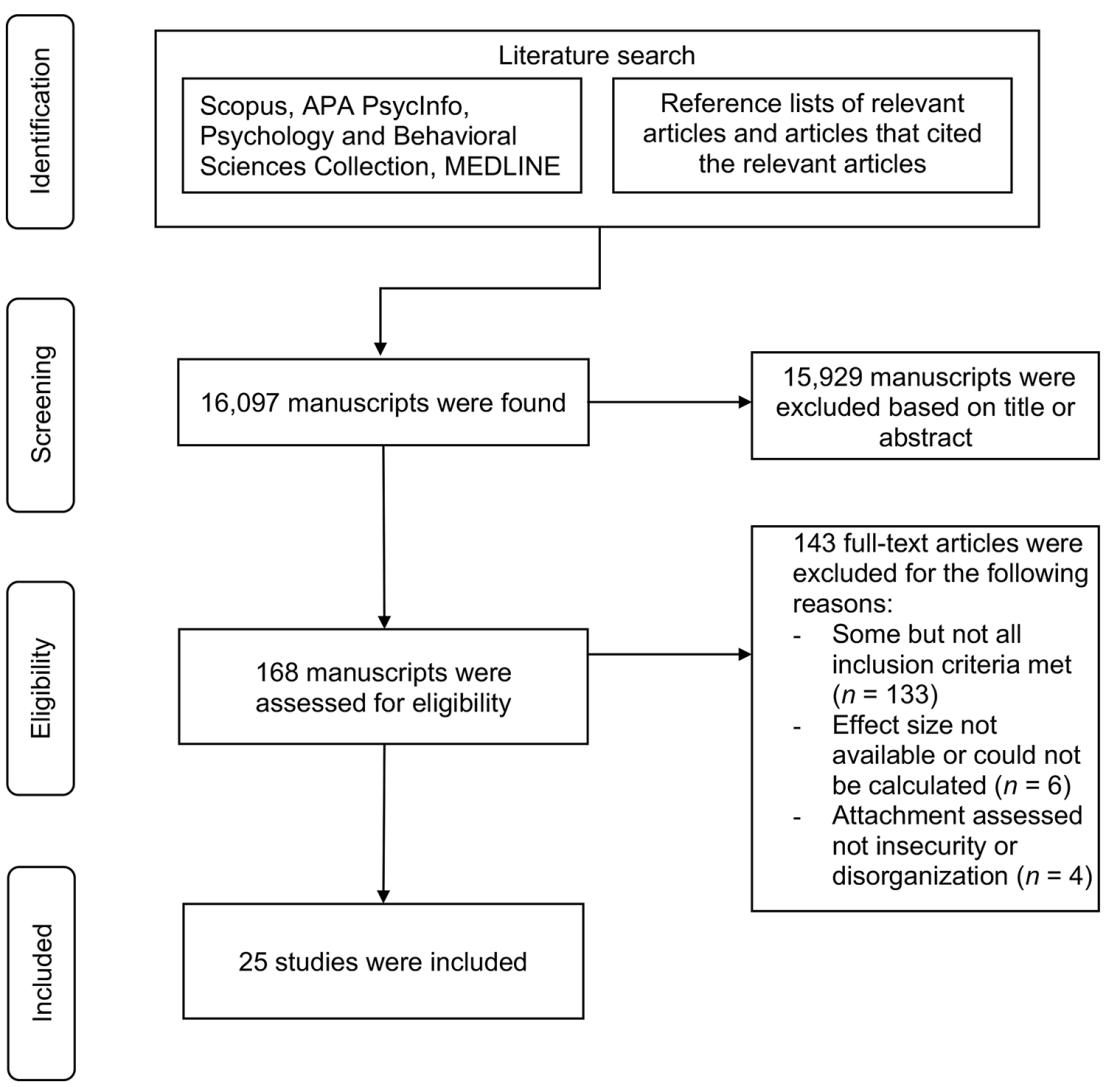
Flow diagram.

Different search strategies were employed to perform the literature search. First, articles published in scientific journals, dissertations, and theses were searched on EBSCO across three electronic databases: MEDLINE, APA PsycInfo, and Psychology and Behavioral Sciences Collection, and on Scopus. We used the following combinations of keywords: (“maltreatment” OR “abuse” OR “neglect” OR “childhood trauma” OR “harsh parent*” OR “harsh discipline” OR “adverse childhood experiences”) AND (“parent–child relationship” OR “mother–child relationship” OR “father–child relationship” OR “parent–child bond” OR “mother–child bond” OR “father–child bond” OR “attachment”). No time period or language restrictions were applied. These searches were conducted in October 2022 and updated in September 2023. The identified references were screened with Rayyan (Ouzzani et al., 2016). Second, we searched the reference lists of all relevant articles that were retrieved with the first search. Third, the studies that cited the relevant articles in Google Scholar were examined. Overall, 16,097 articles were found, screened, and assessed for eligibility by a first coder. A second coder then independently screened 30% (*k* = 4,829) of the articles. Interrater agreement was 99.9% with κ = .80. Disagreements were resolved through discussion, but this did not result in additional studies being included beyond those initially decided upon by the first coder. To determine whether studies were to be included, the coders read titles and abstracts, and if necessary, full article texts. In case of uncertainty regarding the inclusion of a study, the decisions were discussed with the other authors. The study selection process is illustrated in a flowchart (Figure 1).

### Eligibility Criteria

The criteria for including studies in this meta-analysis were the following. First, studies had to include parent–offspring dyads with no restriction of offspring age. Second, they had to report on one or more associations between parental history of CM and offspring attachment security and/or disorganization. Finally, studies had to be available in English or French. The following exclusion criteria were used: (a) an effect size or sufficient information to calculate an effect size was not available (within the manuscript or after contacting the authors), (b) the attachment measure did not assess attachment security or disorganization.

### Coding of Studies

A specific coding system was developed to extract relevant information on potential moderators (see Supplemental Appendix A). The included studies were coded for: offspring mean age, parent and offspring gender distributions, type of variable used to assess CM and attachment, informant of CM and attachment, timing of CM assessment, type of CM assessed, type of attachment measure, measured attachment construct, offspring CM status, parental psychopathology, study country and continent, and publication year. The sociodemographic risk was coded based on Cyr et al.’s (2010) meta-analysis and included the following indicators: low income (identified by the study authors as a low-income sample), parental substance abuse, ethnic minority, single parenthood, low education (12 years of education or less), adolescent parent (20 years or younger when the target child was born). Each indicator was coded 0 if less than one-third of participants corresponded to the indicator, 0.5 if more than one-third but less than two-thirds of participants met the criteria, and 1 if more than two-thirds of the sample met the criteria. Studies that did not report the exact proportion of participants corresponding to the risk indicator were coded 1 if the sample was described as “at risk” for that indicator and 0 if no mention of risk status for that indicator was made. The scores of different indicators were summed to obtain a sociodemographic risk rating ranging from 0 to 6. Finally, we coded the following study quality indicators after selecting and adapting relevant items from section 3 of the Mixed Methods Appraisal Tool (Hong et al., 2018) to fit the aims of this study: (a) sample representativeness of the target population; (b) use of a validated measure for parental CM and attachment; (c) acceptable reliability of the CM and attachment measures (i.e., κ ≥ .5, intraclass correlation coefficient (ICC) ≥ 0.6 or α ≥ .7) or reliability of CM measure by official records; (d) whether there was complete outcome data. Indicators a and d were coded 0 (no or information not available) or 1 (yes), and indicators b and c were coded for the measurement of each construct as follows: 0 if the measure(s) did not correspond to the indicator, 0.5 if at least half of the relevant measures met the indicator criteria (when more than one measure was used to assess the same construct), and 1 if the measure(s) corresponded to the criteria. These scores were summed to obtain an overall quality rating ranging from 0 to 6. When the information on any moderator was missing, it was coded “not specified.” Coding was carried out by one coder, and five (20%) studies were double-coded. The interrater reliability for categorical variables was κ = .96 and for continuous variables ICC = 1.00. Disagreements were resolved by discussion and consensus data were used in the analyses.

### Meta-Analytic Procedures

For each study, we extracted effect sizes that were closest to the raw data and calculated correlation coefficients to quantify the effects of parental history of CM on offspring’s attachment security and disorganization. When necessary, we converted statistical information provided in the study into correlation coefficients using the Practical Meta-Analysis Effect Size Calculator of Wilson (www.campbellcollaboration.org), which is based on Lipsey and Wilson (2001). The effects were coded such that positive correlations reflected positive associations between parental history of CM and attachment insecurity or disorganization. As it has been recommended by a number of researchers (Alexander et al., 1989; Harrer et al., 2022), correlation coefficients were converted following the Fisher *Z* transformation before the statistical analyses to avoid bias related to non-normal distributions of correlations. After performing the analyses, Fisher’s *z*-scores were back-transformed into correlation coefficients to enhance their interpretability. To identify possible outlying effect sizes, we searched for effect sizes that were over 3.29 standard deviations above or below the mean effect size.

As the included studies were considered to constitute a random sample from a population of studies, a random effects meta-analytic approach was used. Furthermore, more than one effect size could be extracted from several studies, as they reported on more than one association between parental history of CM and child attachment (see Supplemental Appendix B for the number of extracted effect sizes per study). This was mainly due to the fact that associations between different types of parental CM and attachment were examined and that attachment was measured as both a categorical and a continuous variable in the same study. Given that effect sizes extracted from an individual study are likely to be more similar than those extracted from different studies (Houben et al., 2015), it is necessary to account for the dependence between effect sizes extracted from the same study (Van den Noortgate et al., 2013). A three-level meta-analytic approach, as described by Assink and Wibbelink (2016), was thus used in the present meta-analyses. In a three-level meta-analytic model, different sources of variance are modeled: level 3 corresponds to the between-study variance, level 2 to the variance between effect sizes within studies (within-study variance), and level 1 to the sampling variance (Van den Noortgate et al., 2013). Hence, effect sizes were not aggregated at the study level but were all entered in the analyses while applying a three-level approach to account for the dependence between them. When different studies reported on the same sample, they were considered as one study (see Supplemental Appendix B).

The statistical analyses were conducted in R version 4.3.0 (R Core Team, 2023) with the metafor (Viechtbauer, 2010), clubSandwich (Pustejovsky, 2022), and dplyr (Wickham et al., 2023) packages. We used the syntax described by Assink and Wibbelink (2016) to estimate the overall effect of parental history of CM, to examine the within- and between-study variances, and to conduct the moderator analyses. Model coefficients were estimated using the restricted maximum likelihood method (Viechtbauer, 2010). The Knapp–Hartung adjustments (Knapp & Hartung, 2003) were applied to calculate the confidence interval around the combined effect. To determine the significance of the within-study (level 2) and between-study (level 3) variances, two separate one-sided log-likelihood ratio tests were conducted as described by Assink and Wibbelink (2016). These tests consist of comparing the fit of the full model (with freely estimated within-study and between-study variances) to the fit of two other models without either within- or between-study variance. For the purpose of moderator analyses, a dummy variable was created for each category of all the categorical variables. Continuous variables were mean-centered.

### Publication Bias

Several statistical methods were used to examine publication bias. First, visual assessment of a funnel plot was conducted. Second, funnel plot asymmetry was assessed with a modified version of the Egger’s regression test (Egger et al., 1997), namely, the “Egger MLMA” test which accounts for the dependence between effect sizes, recommended by Rodgers and Pustejovsky (2021) who demonstrated that ignoring such dependence increases Type I error rates. This analysis was carried out using the code provided by Rodgers and Pustejovsky (2021). Finally, the trim and fill method (Duval & Tweedie, 2000) was applied to adjust for funnel plot asymmetry and to calculate a bias-corrected estimate. Given that the trim and fill method has been shown to produce close to nominal Type I error rates when effect sizes are aggregated at the study level (Rodgers & Pustejovsky, 2021), we conducted the trim and fill test using aggregated effect sizes. Effect sizes were aggregated using the code provided by Viechtbauer (2022).

## Results

### Characteristics of Studies

In total, 25 studies comprising 23 unique samples (*N* = 2,592) and 61 effect sizes were included in the present meta-analyses. Study characteristics are displayed in Supplemental Appendix B.

### Parental History of CM and Offspring Attachment Insecurity

Twenty studies (*N* = 1,950) and 35 effect sizes were included (see Supplemental Appendix B). Two studies were considered as the same study given that they reported on the same sample. On average, 1.75 effect sizes were extracted from each study (*SD* = 1.33, range = 1–6). Offspring average age at the moment of the assessment of attachment security was *M*_weighted_ = 19.15 months, *SD*_weighted_ = 12.59; range = 12–79 months). On average, 49.52% of the offspring participants were women (*SD* = 4.71, *k* = 12, *u* = 18). In one included study, less than 10% of participants were fathers. All other samples were composed of mothers exclusively. In the included samples, the sociodemographic risk index varied from 0 to 4 (*M* = 1.50; *SD* = 1.30). The proportions of parents with psychopathology varied from 20.1% to 100% (*M* = 35%, *SD* = 22.63, *k* = 8, *u* = 14). One study included a sample in which all children were victims of CM (*u* = 4). Included studies were published between 1996 and 2022, with a median publication year of 2017. Study quality ratings ranged from 2 to 5 (*M* = 3.45, *SD* = 1.00). Among the included studies, 14 were conducted in the United States, 2 in Canada, and 1 in each of the following countries: Australia, Germany, Sweden, and Turkey. Grouping studies by continent yielded the following distribution: 16 studies (80%, *u* = 24) were conducted in North America, 2 (10%, *u* = 4) in Europe, 1 (0.5%, *u* = 6) in Australia, and 1 (0.5%, *u* = 1) in West Asia (given that Asia is culturally heterogeneous and the world’s largest continent, it was chosen to indicate the region of the continent).

**Figure 2.**
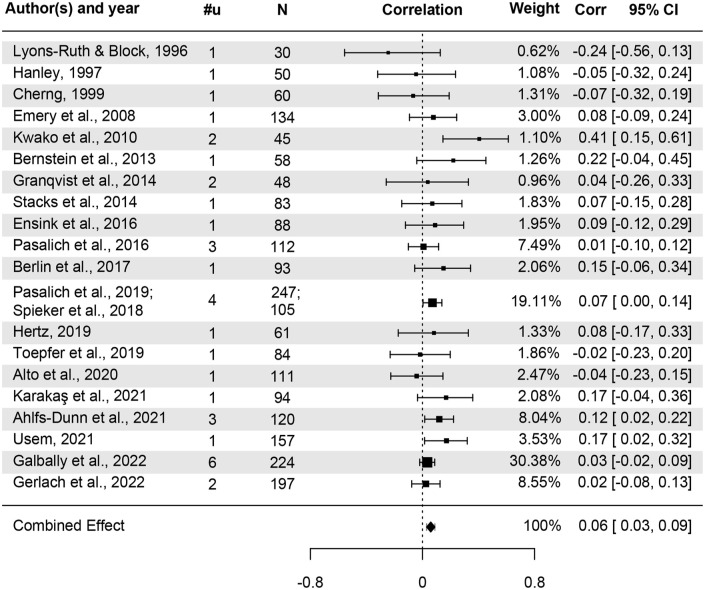
Forest plot of effect sizes for insecurity. *Note.* Corr = Correlation coefficient. Studies are sorted by publication year; #u = Number of effect sizes.

A three-level random effects meta-analysis yielded an estimated overall effect of parental history of CM on attachment insecurity of *r* = .06, *p* < .001, 95% CI [0.03, 0.09]. Figure 2 depicts the combined effect size and the effect sizes aggregated at the study level. One outlying effect size (deviating 3.42 *SD* from the mean) was found. When the analysis was conducted without this outlying effect size, the overall effect remained similar (*r* = .06, *p* < .001, [0.03, 0.09]). The following analyses were conducted with the outlying effect size.

Overall, heterogeneity was null (*I*^2^ = 0.0%). Hence, no significant within- or between-study variance was observed: the two-level models without either within-study or between-study variance showed an equivalent fit compared to the full model (LRT = 0.00, *p* = 1.000 and LRT = 0.00, *p* = 1.000, respectively). Despite null heterogeneity, it was deemed important to test whether study quality and publication year moderated effect sizes. Study quality was not a significant moderator, *F*(1, 33) = 3.40, *p* = .074, and no moderating effect was found for publication year, *F*(1, 33) = 0.27, *p* = .607. Other moderator analyses were also conducted, but no significant moderators were identified (Supplemental Appendix C).

**Figure 3.**
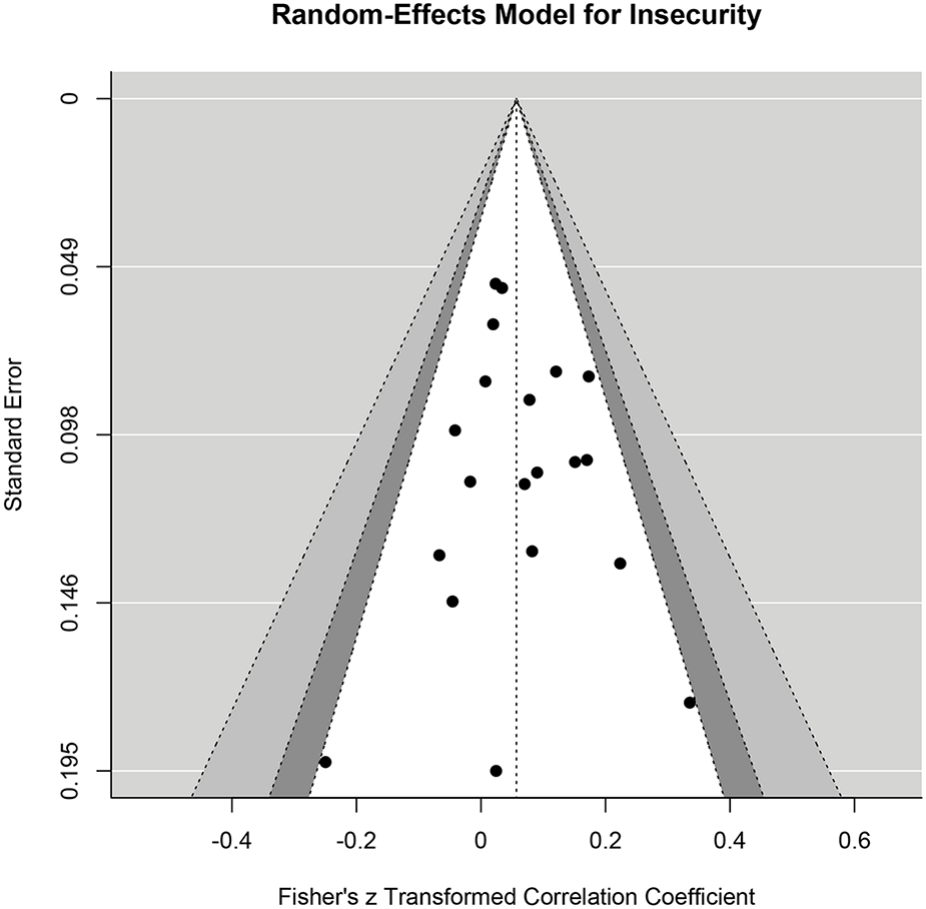
Funnel plot for insecurity. *Note.* Contour-enhanced funnel plot. The black dots depict the observed effect sizes aggregated at the study level. From inside to outside, the dotted lines limit the 90%, 95%, and 99% pseudo-confidence interval regions.

With regard to publication bias, visual inspection of the funnel plot of Fisher’s *z*-scores against standard errors (Figure 3) did not reveal clear asymmetry. Furthermore, the “Egger MLMA” test (Egger et al., 1997; Rodgers & Pustejovsky, 2021) was not significant (β = .74, *p* = .089). Results of the trim-and-fill analysis (Duval & Tweedie, 2000) supported these findings by failing to show evidence of publication bias.

### Parental History of CM and Offspring Attachment Disorganization

A total of 12 studies (*N* = 1,378) and 26 effect sizes examining the link between parental history of CM and offspring attachment disorganization were included in this meta-analysis (see Supplemental Appendix B). Two studies were considered as the same study given that they reported on the same sample. On average, 2.25 effect sizes were extracted from each study (*SD* = 1.86, range = 1–6). Offspring’s average age at the moment of the assessment of attachment was *M*_weighted_ = 18.35 months (*SD*_weighted_ = 19.99, range = 12–79 months). On average, 47.77% of the participants included in samples were girls (*SD* = 6.14, *k* = 9, *u* = 18). All samples were composed of mothers exclusively. In the included samples, the sociodemographic risk index varied from 0 to 4 (*M* = 1.58; *SD* = 1.41). The proportion of parents with psychopathology varied from 9.1% to 100% (*M* = 44.01, *SD* = 30.21, *k* = 7, *u* = 13). None of the studies included child samples exposed to CM. Studies were published between 1996 and 2022, with a median publication year of 2016. Study quality ratings ranged from 2 to 5 (*M* = 3.67, *SD* = 0.98). Six of the included studies were conducted in the United States, four in Canada, one in Australia, and one in Sweden. With regard to the study continent, 10 studies (83%, *u* = 18) were conducted in North America, 1 (0.8%, *u* = 6) in Australia, and 1 (0.8%, *u* = 2) in Europe.

**Figure 4.**
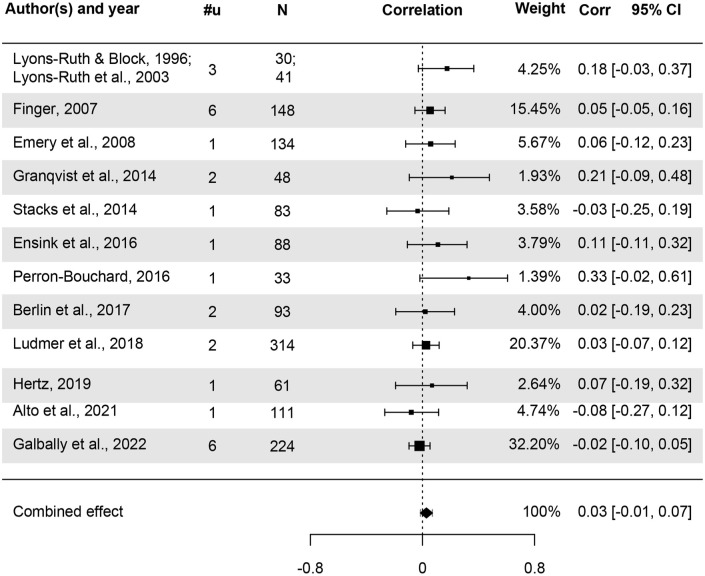
Forest plot of effect sizes for disorganization. *Note*. #u: Number of effect sizes, Corr: Correlation coefficient. Studies are sorted by publication year.

The combined effect of parental history of CM on offspring attachment disorganization, obtained with a three-level random effects meta-analysis, was not significant: *k* = 12, *u* = 26, *r* = .03, *p* = .209, 95% CI [−0.02, 0.07]. The combined effect size and the effect sizes aggregated at the study level are depicted in Figure 4. One outlying effect size (deviating 3.47 *SD* from the mean) was identified. When the analysis was conducted excluding this effect size, the overall effect of parental history of CM on their offspring’s attachment disorganization was *k* = 12, *u* = 25, *r* = .02, *p* = .327, [−0.02, 0.06]. The following analyses were conducted with the outlying effect size.

Overall heterogeneity was *I*^2^ = 7.7%. This was fully attributable to between-study variance. The two-level models without within- and between-study variance showed an equivalent fit compared to the full model (LRT = 0.00, *p* = 1.00, LRT = 0.35, *p* = .552, respectively), indicating no significant within- or between-study variance.

Despite low heterogeneity, study quality and publication year were tested as potential moderators. Study quality did not moderate effect sizes, *F*(1, 24) = 0.91, *p* = .349. Publication year was found to be a significant moderator, *F*(1, 24) = 5.18, *p* = .032. Effect sizes were smaller in studies published more recently (*b* = −0.01, *p* = .032). When the moderating effect of publication year was taken into account, overall heterogeneity was reduced to *I*^2^ = 0.0%. Other moderator analyses were however conducted, but no significant moderators were identified (Supplemental Appendix D).

**Figure 5.**
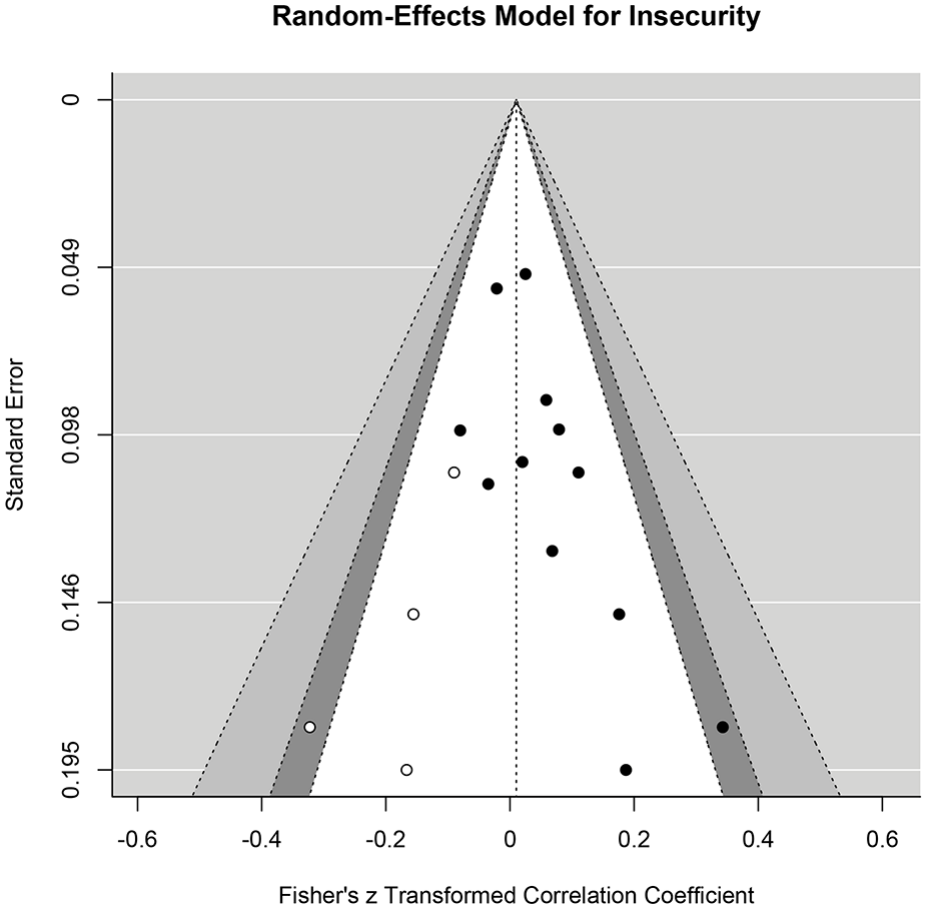
Funnel plot for disorganization. *Note.* Contour-enhanced funnel plot. The black dots depict the observed effect sizes aggregated at the study level, and the white dots depict the imputed effect sizes. From inside to outside, the dotted lines limit the 90%, 95%, and 99% pseudo-confidence interval regions.

With regard to publication bias, visual assessment of the funnel plot (Figure 5) indicated the presence of publication bias. The “Egger MLMA” test (Egger et al., 1997; Rodgers & Pustejovsky, 2021) of funnel plot asymmetry was also significant (β = 1.36, *p* < .001), confirming a potential publication bias. Results of the trim-and-fill analysis (Duval & Tweedie, 2000) indicated that four effect sizes had to be added on the left side to reach funnel plot symmetry (Figure 5) and indicated a corrected combined non-significant effect of *r* = .01, *p* = .677, 95% CI [−0.04, 0.06].

## Discussion

The present meta-analyses were aimed at synthesizing available studies that document associations between parental history of CM and offspring attachment insecurity and disorganization. In the first meta-analysis, we found a weak but significant and positive combined effect size for the association between parental history of CM and child attachment insecurity. The second meta-analysis on attachment disorganization revealed a non-significant combined effect size close to zero. In the two meta-analyses, heterogeneity was, respectively, null and low, indicating that effect sizes were highly consistent within and across studies.

The hypothesis of a positive association between parental history of CM and offspring attachment insecurity was, to some extent, supported. However, the combined effect size that was found indicated that parental history of CM is only weakly associated with offspring insecure attachment. This suggests that children whose parents have been exposed to CM may be at a slightly higher risk of developing insecure attachment. Consequently, factors other than parental childhood trauma seem more likely to explain alterations in parenting quality which is associated with attachment (in)security (e.g., Madigan et al., 2024). In this regard, more proximal risk factors, which have been associated with attachment insecurity, such as socioeconomic risk (e.g., see Cyr et al., 2010; Madigan et al., 2023 for meta-analyses), parental conflict (e.g., Brock & Kochanska, 2016), offspring exposure to CM (see Cyr et al., 2010 for meta-analysis), and intimate partner violence between parents (see McIntosh et al., 2021 for meta-analysis) may be more determining than parents’ history of CM.

Furthermore, although we expected parental history of CM to be associated with offspring attachment disorganization as well, the combined effect of parental history of CM on disorganized child attachment was close to zero and non-significant. This suggests that despite the negative effects of CM, which may persist into adulthood (e.g., see Baldwin et al., 2023 for meta-analysis), children of parents who report CM are not more likely to show disorganized attachments than those of parents who do not report CM. Like for insecure attachment, factors other than parental history of CM are thus at play. By altering the quality of parenting, they could affect attachment organization in the second generation. Previous research has pointed to factors such as socioeconomic risk (e.g., see Cyr et al., 2010; Madigan et al., 2023 for meta-analyses), offspring CM (see Cyr et al., 2010 for meta-analysis), and parent’s unresolved attachment state of mind (see Verhage et al., 2016 for meta-analysis) as significantly increasing chances of disorganized attachment in children.

Based on research showing a significant correspondence between parental unresolved state of mind regarding past abuse (or loss) and child attachment disorganization (see Verhage et al., 2016 for meta-analysis), it could be argued that it is the unresolved state of mind following abuse that is related to disorganized attachment in the child rather than abuse itself. This hypothesis seems reasonable given that mothers with histories of CM who are classified as unresolved with respect to abuse are likely to show more abuse-related post-traumatic stress disorder (PTSD) and more PTSD avoidant symptoms than those who are not unresolved (Stovall-McClough & Cloitre, 2006). Such symptoms may, in turn, alter parenting (see Christie et al., 2019 for systematic review). Furthermore, research findings also indicate that resolution of trauma moderates the effect of neglect on maternal sensitivity (Swerbenski et al., 2023) and that better maternal trauma-related reflective functioning is associated with less disorganized attachments in children (Berthelot et al., 2015). Hence, the experience of abuse may impact the parent–child relationship and subsequent offspring attachment only when it remains unresolved or not reflected upon. Research on the moderating role of unresolved trauma in the link between parental CM and offspring attachment is needed.

Overall, findings from this study lend little support to the hypothesis that the deleterious effects of CM are transmitted to the next generation and would result in less optimal child attachment. This suggests a rather non-deterministic view of childhood relational trauma with regard to the parent–child relationship in the second generation: CM in itself does not appear to significantly impact the parents’ ability to build secure relationships with their children.

### Assessment of CM

When interpreting the findings of this study, it is necessary to consider that most studies focused on a combination of different types of CM or CM more generally, rather than on specific types of CM. When moderator analyses were conducted, only one comparison was possible due to the small number of studies focusing on specific types of CM. For the meta-analysis on insecurity, effect sizes on global CM and sexual abuse were compared (given that at least four studies reported on these associations). This comparison was non-significant. However, given the small number of effect sizes extracted for specific types of CM, findings from the current study should be interpreted with caution. It cannot be excluded that associations of different strengths would be found between specific forms of CM and offspring attachment.

In addition, it should be noted that retrospective and self-report measures were used in all but one of the included studies. Hence, it is important to acknowledge that biases related to these types of measures (Baldwin et al., 2019; Cooley et al., 2022; Negriff et al., 2017) may have affected effect sizes across studies. This appears particularly relevant given that the findings of Bernstein et al. (2013) showed that parents who have been exposed to childhood trauma and who idealize their own parents are more likely to have insecure-avoidant children than parents who do not idealize their maltreating parents. Moreover, as noted by Baldwin et al. (2019), prospective and retrospective measures of CM identify largely different groups of individuals. Motivational issues and memory biases are argued to reduce agreement between these two types of measures, some individuals being prone to underreporting CM while others may overreport such experiences (Baldwin et al., 2019). Therefore, findings from the present meta-analyses should be interpreted as relevant to self-reported victims of CM only, that is, individuals who recalled CM and were willing to report it.

Together these considerations point to the importance of interpreting the findings of the present meta-analyses with caution. More investigations using prospective measures and multiple informants of CM, and distinguishing different types of CM are needed before concluding that parental history of CM has little or no effect on offspring attachment.

### Potential Moderators and Homogeneity

In addition to methodological features regarding CM assessment, we also aimed at examining other potential moderators of the association between parental history of CM and offspring attachment. However, effect sizes were found to be consistent within and across studies. Despite null heterogeneity, moderator analyses were conducted. They revealed that publication year moderated effect sizes in the meta-analysis on attachment disorganization, but no other significant moderators were found in either meta-analysis.

The moderating effect of publication year suggests that the intergenerational impact of parental CM on offspring disorganized attachment tends to diminish with time. This may be partly related to changes in how CM is perceived (Spilsbury et al., 2018) and to the evolutions of government policies regarding CM, such as expanded definitions of maltreatment and policies aimed at better recognizing and managing it (Gilbert et al., 2012). Over the years, these changes may have helped improve the detection and reporting of CM, allowing for interventions to be proposed to a larger number of victims, and potentially, thereby reducing the intergenerational impact of CM. On the other hand, the moderating effect of publication year could also indicate the presence of a decline effect, possibly related to publication bias (Schooler, 2011), which will be discussed below.

It must be mentioned that there was little or no between- or within-study variation with regard to a number of substantive and methodological moderators. As for substantive moderators, it is to note that virtually all participants in the included studies were mothers. Two samples included in the meta-analysis on attachment insecurity comprised fathers: they represented less than 10% (*n* < 25) and 2.5% (*n* = 5) of Pasalich et al. (2019) and Spieker et al.’s (2018) and Gerlach et al.’s (2022) samples, respectively. Therefore, less than 30 out of the 1,950 parents of the samples included in this meta-analysis were fathers. Moreover, offspring’s mean age ranged from 12 to 79 months in the included studies. With regard to offspring maltreatment status, only one sample, included in the meta-analysis on attachment insecurity, comprised offspring victims of CM (Pasalich et al., 2019; Spieker et al., 2018), while no offspring CM samples were included in the one on disorganization. Consequently, findings from the current meta-analyses must be considered as pertaining to mother–child dyads including infants and young children not exposed to CM.

Regarding the assessment of attachment, external observers were used as informants in all the included studies. Furthermore, relationship-specific and behavioral rather than general representational measures of offspring attachment were used in all the studies except for two: those of Kwako et al. (2010; *n* = 20; included in the meta-analysis on insecurity) and of (Granqvist et al. 2014; *n* = 40; included in the two meta-analyses) who used a representational measure to assess the general attachment model of participating children. No attachment questionnaire was used in any of the included studies. Finally, there was little variability with regard to the geographic region of the studies, with most of the included studies conducted in North America. Of the 20 studies included in the meta-analysis on attachment insecurity, 2 were conducted in Europe, 1 in West Asia, and 1 in Australia while the others were conducted in North America. Among the 12 studies included in the meta-analysis on attachment disorganization, 1 was conducted in Australia, 1 in Europe, and the rest in North America. This is consistent with Henrich et al.’s (2010) observation that most behavioral research is conducted on samples drawn from Western, Educated, Industrialized, Rich, and Democratic (WEIRD) societies, which raises important issues of generalizability. With regard to the present meta-analyses, it appears plausible that notable differences exist between countries and cultures in the ways CM is defined, viewed, and responded to, as well as in who the children’s primary caregivers are (see Van IJzendoorn et al., 2006). Therefore, the association between parental exposure to CM and offspring attachment could be expected to differ from one country/culture to another. The moderator effect of study continent could not however be conducted given the small number of studies conducted on continents other than North America. Hence, findings from the current meta-analyses should be interpreted as mainly relevant to the North American context. More research on this topic should be conducted outside North America or WEIRD countries more generally.

Included studies varied with respect to other characteristics. Nevertheless, effect sizes were found to be consistent across studies for both attachment insecurity and disorganization, suggesting that maternal history of CM has little or no effect on offspring attachment regardless of whether studies included continuous or categorical measures of CM or attachment, participants with lower or higher sociodemographic risk status, parental psychopathology, and regardless of offspring gender and age, as well as study quality. It is interesting to note that maternal history of CM does not appear more likely to impact offspring attachment in families that are at elevated sociodemographic risk, even though increased proportions of insecure attachment and disorganization have been found in such contexts (see Cyr et al., 2010; Madigan et al., 2023 for meta-analyses). Consequently, maternal history of CM may not add to the risk of insecure or disorganized attachment associated with sociodemographic risk.

### Limitations

The present study has limitations that must be acknowledged. Some of these limitations have been described previously, namely: the vast majority of included studies were conducted in North America, did not document the effect of specific types of CM, were based on self-report retrospective measures of CM, included virtually only mothers and exclusively infants and young children. These limitations affect the generalizability of our findings. Furthermore, evidence of publication bias was found for the meta-analysis on attachment disorganization, possibly implying that the validity of the overall effect found is questionable. Yet the overall effect remained non-significant, after four effect sizes were imputed with the Duval and Tweedie’s (2000) trim and fill method to compensate for publication bias. Finally, the quality of the included studies varied from 2 to 5 on a 7-point scale suggesting that findings from some included studies may have been subject to some level of bias. However, moderator analyses showed that effect sizes did not vary as a function of study quality.

### Conclusion

Despite these limitations, this study contributes to enhancing the understanding of the effects of CM on the next generation. Namely, by suggesting that maternal history of CM (all types confounded) is rather unlikely to notably impact child attachment, our findings provide a much more nuanced view of the consequences of maternal history of CM on the parent–child relationship. It appears that women who report having been exposed to such events are not at particular risk of failing to establish a secure relationship with their children. However, research including fathers and prospective and objective measures of parental exposure to CM is needed to gain a more comprehensive view of the possible intergenerational effects of CM on offspring attachment. Furthermore, given the inherently interpersonal nature of CM trauma, examining whether and to what extent proximal interpersonal factors (such as the quality of the relationship between parents, or the quality of the relationship between the other parent and offspring) potentiate or mitigate the effect of parent’s history of CM on the future generation is an interesting avenue for future research.

**Table:** Summary of Critical Findings.

• Mothers’ self-reported history of childhood maltreatment was weakly related to insecure and not related to disorganized attachment in offspring.
• Effect sizes were consistent across and within studies.
• Parental childhood maltreatment was assessed with retrospective self-report measures in almost all the studies.
• Very few studies on the intergenerational links between parental childhood maltreatment and offspring attachment include fathers or assess specific types of maltreatment.

**Table:** Implications for Research, Policy, and Practice.

Research
• Research with prospective and multi-informant measures of parental exposure to childhood maltreatment is needed.
• Links between specific types of parental childhood maltreatment and offspring attachment still need to be examined.
Policy and practice
• Research findings suggest that it is the non-resolution (i.e., integration) of parental childhood maltreatment, rather than childhood maltreatment itself, that is associated with disorganized offspring attachment.
• Efforts should be made to reduce the stigma associated with victimization and to encourage adult victims to seek help to resolve trauma resulting from childhood maltreatment.

## Supplemental Material

sj-docx-1-tva-10.1177_15248380241282995 – Supplemental material for Parental History of Childhood Maltreatment and Offspring Attachment Insecurity and Disorganization: Two Meta-Analyses Supplemental material, sj-docx-1-tva-10.1177_15248380241282995 for Parental History of Childhood Maltreatment and Offspring Attachment Insecurity and Disorganization: Two Meta-Analyses by Aino Elina Sirparanta, Camille Danner Touati, Chantal Cyr and Rapha00EBle Miljkovitch in Trauma, Violence, & Abuse
