# Supplementary material for: Parental History of Childhood Maltreatment and Offspring Attachment Insecurity and Disorganization: Two Meta-Analyses
Source: Trauma Violence Abuse. 2024 Oct 1;26(3):420–35. doi: 10.1177/15248380241282995 (PMC12145480; doi:10.1177/15248380241282995)
Supplement: sj-docx-1-tva-10.1177_15248380241282995 – Supplemental material for Parental History of Childhood Maltreatment and Offspring Attachment Insecurity and Disorganization: Two Meta-Analyses [file sj-docx-1-tva-10.1177_15248380241282995.docx]

**Appendices**

**Appendix A**

*Coding System of the Variables Extracted for Analyses*

| Variable | Coding |
| --- | --- |
| Substantive moderators |  |
| Offspring age | Offspring mean age in months |
| Offspring gender | Percentage of girls in offspring sample |
| Parent gender | Percentage of mothers |
| Offspring CM | Percentage of offspring exposed to CM |
| Parent psychopathology | Percentage of parents with psychopathology |
| Sociodemographic risk | Low income  Parental substance abuse  Ethnic minority  Single parenthood  Low education (≤ 12 years)  Adolescent parent (≤ 20 years)  Coding: 0 = < 0.33% of sample corresponded to indicator;  0.5 = 0.33%-0.67% of sample corresponded to indicator;  1 = > 0.67% of sample corresponded to indicator  Overall rating: sum of individual ratings |
| Methodological moderators |  |
| CM: type of variable | Categorical  Continuous |
| CM: assessment timing | Prospective  Retrospective |
| CM: informant | Self-report  External  Multiple |
| CM: type | Global score  Physical abuse  Sexual abuse  Emotional abuse  Neglect |
| Attachment: type of variable | Categorical  Continuous |
| Attachment: type of measure | Behavioral  Representational  Questionnaire |
| Attachment: construct | General trait  Relationship-specific |
| Attachment: informant | Self-report  External  Caregiver |
| Publication year | Study publication year |
| Study country and continent | Country in which the study was conducted  Continent on which the study was conducted |
| Study quality | Participants representative of target population;  Use of a validated measure for parental CM;  Acceptable reliability of the CM measure (i.e., κ = 0.5, ICC = 0.6 or α = 0.7) or reliability of measure by official records;  Use of a validated measure for attachment;  Acceptable reliability of the attachment measure (i.e., κ = 0.5, ICC = 0.6 or α = 0.7);  Complete outcome data;  Coding: 0 = did not correspond to the indicator or information not available;  0.5 = at least half of the relevant measures corresponded to the indicator (when more than one measure was used to assess the same construct);  1 = corresponded to the indicator  Overall rating: sum of individual ratings |

**Appendix B**

Studies Included in the Meta-Analysis

|  |  |  | Offspring | | Measures | |  |  | #*u* | |
| --- | --- | --- | --- | --- | --- | --- | --- | --- | --- | --- |
| Studies | Country | *N* | % F | Age | CM | Att | Risk Index | Study Qual. | I | D |
| Ahlfs-Dunn et al., 2022 | USA | 120 | - | 12 | CTQ | AQS | 2.5 | 3 | 3 | 0 |
| Alto et al., 2021 | USA | 111 | 49 | 26 | CTQ | SSP | 3.5 | 5 | 1 | 1 |
| Berlin et al., 2017 | USA | 93 | 50 | 17 | CTSPC adapted | SSP | 3 | 3 | 1 | 1 |
| Bernstein et al., 2013 | USA | 58 | 32.6 | 18 | BBTS | SSP | 1.5 | 3 | 1 | 0 |
| Cherng, 1999 | USA | 60 | - | 12 | PPSAE III + interview | SSP | 1 | 2 | 1 | 0 |
| Emery et al., 2008 | Canada | 134 | 42.9 | 15 | CTQ CSAI | SSP | 4 | 5 | 1 | 1 |
| Ensink et al., 2016 | Canada | 88 | 50 | 16 | Phone interview + AAI | SSP | 0 | 4 | 1 | 1 |
| Finger, 2007 | USA | 148 | 52 | 15 | CTQ | SSP | 1.5 | 2 | 0 | 6 |
| Galbally et al., 2022 | Australia | 224 | - | 12 | CTQ | SSP | 0 | 4 | 6 | 6 |
| Gerlach et al., 2022 | Germany | 197 | 50 | 15; 22 | KiD | AQS | 0 | 4 | 2 | 0 |
| Granqvist et al., 2014 | Sweden | 48 | 57 | 79 | iATM | SAT | 2; 3 | 5 | 2 | 2 |
| Hanley, 1997 | USA | 50 | 52 | 56 | CMIS-SF | Modified SSP | 0 | 5 | 1 | 0 |
| Hertz, 2019 | USA | 61 | - | 12 | CTQ | SSP | 1 | 3 | 1 | 1 |
| Karakaş et al., 2021 | Turkey | 94 | - | 13 | ACE-Q | SSP | 0 | 2 | 1 | 0 |
| Kwako et al., 2010 | USA | 35 | 50 | 55 | Reports | SSP  PAA  SAA | 1 | 3 | 2 | 0 |
| Ludmer et al., 2018 | Canada | 314 | 48 | 17 | CTQ-SF | SSP | 0.5 | 4 | 0 | 2 |
| Lyons-Ruth et al., 2003; Lyons-Ruth & Block, 1996 | USA  USA | 30  41 | -  60 | 12; 18 | Adapted AAI  Adapted AAI | SSP  SSP | 1.5  1.5 | 3  3 | 0  1 | 2  1 |
| Pasalich et al., 2016 | USA | 112 | 54 | 12 | Interview | SSP | 3.5 | 4 | 3 | 0 |
| Pasalich et al., 2019;  Spieker et al., 2018 | USA  USA | 247105 | -  - | 16 24 | CTQ-SF  CTQ-SF | TAS-45  TAS-45 | 1.5; 2 | 3  3 | 3  1 | 0  0 |
| Perron-Bouchard, 2016 | Canada | 33 | - | 17 | Adapted AAI | SSP | 0.5 | 3 | 0 | 1 |
| Stacks et al., 2014 | USA | 83 | 41 | 16 | CTQ | SSP | 0 | 3 | 1 | 1 |
| Toepfer et al., 2019 | USA | 84 | - | 12 | CTQ | SSP | 0.5 | 2 | 1 | 0 |
| Usem, 2021 | USA | 157 | 51 | 17 | ACE-Q | SSP | 1.5 | 3 | 1 | 0 |
| *Note*. % F: Percentage of female participants; #*u*: Number of effect sizes; AAI: Adult Attachment Interview; ACE-Q: Adverse Childhood Experiences Questionnaire; AQS: Attachment Q-Sort; Att: Attachment; BBTS: Brief Betrayal Trauma Survey; CM: Child maltreatment; CMIS-SF: Child Maltreatment Interview Schedule – Short Form; CSAI: Childhood Sexual Abuse Interview; CTQ: Childhood Trauma Questionnaire; CTQ-SF: Childhood Trauma Questionnaire – Short Form; CTSPC: Parent-Child Conflict Tactics Scale; D: Meta-analysis on disorganization; I: Meta-analysis on insecurity; iATM: interview for Abuse, Trauma, and Maltreatment; KiD: Kinder in Deutschland; PAA: Preschool Assessment of Attachment; PPSAE III: Physical Punishment Scale for Assessing Environments III; Qual.: Quality; SAA: School-age Assessment of Attachment; SAT: Separation Anxiety Test; SSP: Strange Situation Procedure; TAS-45: Toddler Attachment Sort-45. | | | | | | | | | | |

**Appendix C**

*Results of Moderator Analyses for Insecurity*

| Moderator | #*k* | #*u* | Mean *r* | *b* (*SE*) | *t-*value | *F*(*df*1,*df*2) | *p* |
| --- | --- | --- | --- | --- | --- | --- | --- |
| Substantive moderators |  |  |  |  |  |  |  |
| Offspring age | 20 | 35 |  | 0.00 (0.00) | 1.14 | *F*(1,33) = 1.29 | .392 |
| Offspring gender (% female) | 12 | 18 |  | -0.00 (0.00) | -1.27 | *F*(1,16) = 1.62 | .221 |
| Parent gender (% female) | Not applicable (two samples included < 10% and 2.5% fathers) | | | | | | |
| Offspring CM | Not applicable | | | | | | |
| Non-CM sample | 19 | 31 |  |  |  |  |  |
| CM sample | 1 | 4 |  |  |  |  |  |
| Parent psychopathology (%) | 8 | 14 |  | 0.00 (0.00) | 0.96 | *F*(1,12) = 0.92 | .356 |
| Sociodemographic risk | 20 | 35 |  | 0.01 (0.00) | 0.61 | *F*(1,33) = 0.38 | .544 |
| Methodological moderators |  |  |  |  |  |  |  |
| Assessment of CM |  |  |  |  |  |  |  |
| Type of variable |  |  |  |  |  | *F*(1,33) = 2.08 | .159 |
| Categorical | 11 | 21 | .04 |  |  |  |  |
| Continuous | 10 | 14 | .09 | 0.05 (0.03) | 1.44 |  |  |
| Timing | Not applicable | | | | | | |
| Prospective | 1 | 2 |  |  |  |  |  |
| Retrospective | 19 | 33 |  |  |  |  |  |
| Informant | Not applicable | | | | | | |
| Self-report | 19 | 33 |  |  |  |  |  |
| External | 1 | 2 |  |  |  |  |  |
| Multiple | 0 | 0 |  |  |  |  |  |
| Type of CM (global vs. sexual abuse) |  |  |  |  |  | *F*(1,33) = 0.06 | .801 |
| Global | 17 | 20 | .06 |  |  |  |  |
| Sexual abuse | 6 | 8 | .04 | 0.01 (0.04) | 0.25 |  |  |
| Physical abuse | 3 | 3 | Not applicable | | | | |
| Emotional abuse | 2 | 2 | Not applicable | | | | |
| Neglect | 1 | 2 | Not applicable | | | | |
| Assessment of attachment |  |  |  |  |  |  |  |
| Type of variable |  |  |  |  |  | *F*(1,33) = 1.22 | .277 |
| Categorical | 14 | 21 | .04 |  |  |  |  |
| Continuous | 6 | 14 | .08 | 0.03 (0.03) | 1.10 |  |  |
| Type of measure | Not applicable | | | | | | |
| Behavioral | 18 | 31 |  |  |  |  |  |
| Representational | 2 | 4 |  |  |  |  |  |
| Questionnaire | 0 | 0 |  |  |  |  |  |
| Type of construct | Not applicable | | | | | | |
| General trait | 2 | 4 |  |  |  |  |  |
| Relationship-specific | 18 | 31 |  |  |  |  |  |
| Informant | Not applicable | | | | | | |
| Self-report | 0 | 0 |  |  |  |  |  |
| External | 20 | 35 |  |  |  |  |  |
| Caregiver | 0 | 0 |  |  |  |  |  |
| Study continent | Not applicable | | | | | | |
| North America | 16 | 24 |  |  |  |  |  |
| Europe | 2 | 4 |  |  |  |  |  |
| Australia | 1 | 6 |  |  |  |  |  |
| West Asia | 1 | 1 |  |  |  |  |  |
| Publication year | 20 | 35 |  | 0.00 (0.00) | 0.52 | *F*(1,33) = 0.27 | .607 |
| Study quality | 20 | 35 |  | -0.04 (0.02) | -1.84 | *F*(1,33) = 3.40 | .074 |
| *Note*. #*k*: Number of studies; #*u*: Number of effect sizes. | | | | | | | |

**Appendix D**

*Results of Moderator Analyses for Disorganization*

| Moderator | #*k* | #*u* | Mean *r* | *b* (*SE*) | *t*-value | *F*(*df*1,*df*2) | *p* |
| --- | --- | --- | --- | --- | --- | --- | --- |
| Substantive moderators |  |  |  |  |  |  |  |
| Offspring age | 12 | 26 |  | 0.00 (0.00) | 1.29 | *F*(1,24) = 1.68 | .208 |
| Offspring gender (% female) | 9 | 18 |  | 0.00 (0.01) | 0.52 | *F*(1,16) = 0.27 | .613 |
| Parent gender (% female) | Not applicable (all samples 100% mothers) | | | | | | |
| Offspring CM | Not applicable | | | | | | |
| Non-CM sample | 12 | 26 |  |  |  |  |  |
| CM sample | 0 | 0 |  |  |  |  |  |
| Parent psychopathology (%) | 7 | 13 |  | 0.00 (0.00) | 1.10 | *F*(1,11) = 1.21 | .296 |
| Sociodemographic risk | 12 | 26 |  | 0.02 (0.02) | 0.94 | *F*(1,24) = 0.88 | .358 |
| Methodological moderators |  |  |  |  |  |  |  |
| Assessment of CM |  |  |  |  |  |  |  |
| Type of variable |  |  |  |  |  | *F*(1,24) = 2.10 | .160 |
| Categorical | 4 | 9 | -.01 |  |  |  |  |
| Continuous | 9 | 17 | .04 | 0.05 (0.04) | 1.45 |  |  |
| Timing | Not applicable | | | | | | |
| Prospective | 0 | 0 |  |  |  |  |  |
| Retrospective | 12 | 26 |  |  |  |  |  |
| Informant | Not applicable | | | | | | |
| Self-report | 12 | 26 |  |  |  |  |  |
| External | 0 | 0 |  |  |  |  |  |
| Multiple | 0 | 0 |  |  |  |  |  |
| Type of CM | Not applicable | | | | | | |
| Global | 10 | 14 |  |  |  |  |  |
| Sexual abuse | 2 | 6 |  |  |  |  |  |
| Physical abuse | 2 | 3 |  |  |  |  |  |
| Emotional abuse | 1 | 1 |  |  |  |  |  |
| Neglect | 1 | 2 |  |  |  |  |  |
| Assessment of attachment |  |  |  |  |  |  |  |
| Type of variable |  |  |  |  |  | *F*(1,24) = 0.88 | .357 |
| Categorical | 9 | 13 | .01 |  |  |  |  |
| Continuous | 6 | 13 | .04 | 0.04 (0.04) | 0.94 |  |  |
| Type of measure | Not applicable | | | | | | |
| Behavioral | 11 | 24 |  |  |  |  |  |
| Representational | 1 | 2 |  |  |  |  |  |
| Questionnaire | 0 | 0 |  |  |  |  |  |
| Type of construct | Not applicable | | | | | | |
| General trait | 1 | 2 |  |  |  |  |  |
| Relationship-specific | 11 | 24 |  |  |  |  |  |
| Informant | Not applicable | | | | | | |
| Self-report | 0 | 0 |  |  |  |  |  |
| External | 12 | 26 |  |  |  |  |  |
| Caregiver | 0 | 0 |  |  |  |  |  |
| Study continent | Not applicable | | | | | | |
| North America | 10 | 18 |  |  |  |  |  |
| Australia | 1 | 6 |  |  |  |  |  |
| Europe | 1 | 2 |  |  |  |  |  |
| Publication year | 12 | 26 |  | -0.01 (0.00) | -2.28 | *F*(1,24) = 5.18 | .032 |
| Study quality | 12 | 26 |  | -0.02 (0.02) | -0.96 | *F*(1,24) = 0.91 | .349 |
| Note. #k: Number of studies; #u: Number of effect sizes. | | | | | | | |
